# Engineered Cas12j‐8 is a Versatile Platform for Multiplexed Genome Modulation in Mammalian Cells

**DOI:** 10.1002/advs.202502593

**Published:** 2025-06-10

**Authors:** Ru Meng, Jiayao Li, Wuke Wang, Dong Liang, Zhanwei Li, Cong Mao, Qingyang Li, Yu Zhang, Hao Chen, Jin Tang, Ping Hu, Qi Niu, Xingxu Huang, Bin Shen, Jun Zhang

**Affiliations:** ^1^ State Key Laboratory of Reproductive Medicine and Offspring Health Nanjing Medical University Nanjing Jiangsu 211166 China; ^2^ The Key Laboratory of Pancreatic Diseases of Zhejiang Province The First Affiliated Hospital Zhejiang University School of Medicine Hangzhou Zhejiang 310003 China; ^3^ Department of Prenatal Diagnosis Women's Hospital of Nanjing Medical University Nanjing Women and Children's Healthcare Hospital Nanjing Jiangsu 210004 China; ^4^ Hangzhou LUCA Intelligent Technology Co. Ltd. Hangzhou Zhejiang 310029 China; ^5^ Department of Rare Diseases The First Affiliated Hospital of Nanjing Medical University Nanjing Jiangsu 210029 China

**Keywords:** CRISPR‐Cas12j‐8, multiplexed editing, protein engineering

## Abstract

Cas12j‐8 is a compact Cas nuclease discovered from the metagenome of giant bacteriophages, consisting of only 717 amino acids and recognizing the ‘5‐TTN‐3′ protospacer adjacent motif (PAM) sequence. However, its low gene editing efficiency in mammalian cells limits its application in therapeutic gene editing. To address this limitation, structure‐guided mutagenesis is employed to replace key negatively charged residues with arginine, strengthening DNA binding. The resulting quintuple mutant, engineered Cas12j‐8 (enCas12j‐8), demonstrates robust on‐target editing efficiency comparable to LbCas12a while maintaining low off‐target effects. Cytosine base editors (CBEs) and adenine base editors (ABEs) are developed using enCas12j‐8, achieving up to 29.54‐fold C‐to‐T and 36.57‐fold A‐to‐G conversion efficiency compared with the wild‐type at the dominated sites, respectively. Notably, enCas12j‐8 enables multiplexed editing of three genomic loci simultaneously via a single crRNA array, achieving efficiencies comparable to single‐guide approaches. Additionally, enCas12j‐8‐ABE facilitates the disruption of splice acceptor sites, effectively inducing exon skipping in the SOD1 gene. This strategy holds potential significance for therapeutic genome modulation. These findings establish enCas12j‐8 as a versatile, high‐precision tool for genome engineering, combining efficient delivery, multiplexing capability, and compatibility with diverse editing modalities.

## Graphical abstract

Engineered through structure‐guided protein engineering, enCas12j‐8 significantly enhances the editing efficiency of compact Cas12j‐8 while maintaining high specificity. It enables efficient multiplexed genome editing and base editing using a single crRNA array, demonstrating broad applicability and therapeutic potential in genome engineering.

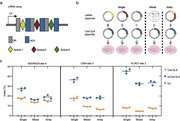

## 1 Introduction

Clustered regularly interspaced short palindromic repeats‐Cas (CRISPR‐Cas) systems function as prokaryotic adaptive immunity in bacteria and archaea, defending against viral infections and plasmid transformation.^[^ ^1^, ^2^ ^]^ Over the past decade, these systems have been repurposed into versatile genome‐editing technologies, with particular advancements in Type II Cas9 and Type V Cas12 systems.^[^ ^3^, ^4^, ^5^ ^]^ Unlike Cas9, which requires an additional RNase III protein for precursor crRNA (pre‐crRNA) processing and a separate trans‐activating crRNA (tracrRNA) to facilitate target DNA cleavage, Cas12a and Cas12i, identified in prokaryotic cells, exhibit intrinsic RNase activity that enables autonomous processing of pre‐crRNA into multiple functional crRNAs.^[^ ^6^, ^7^, ^8^ ^]^ This intrinsic RNase activity makes Cas12a highly amenable to engineering, establishing it as a robust platform for multiplex gene editing. It enables efficient simultaneous editing or transcriptional regulation of multiple genes in mammalian cells.^[^ ^9^, ^10^, ^11^, ^12^ ^]^

Recent metagenomic data mining has identified a series of phage‐encoded CRISPR‐Cas systems, among which Type V members Cas12j(Φ) and Casλ exhibit RuvC nuclease domains structurally analogous to Cas12a.^[^ ^13^, ^14^, ^15^ ^]^ These compact systems mediate both RNA‐guided double‐stranded DNA cleavage and intrinsic pre‐crRNA self‐processing functionalities, yet they are approximately half the size of Cas12a or Cas9. This minimized architecture crucially endows Cas12j/Casλ with enhanced compatibility for multiplex genome‐editing applications and viral vector‐mediated delivery platforms. Notably, Cas12j homologs Cas12j2 and Cas12j‐8 exhibit strong genome‐editing capabilities in plant systems.^[^ ^16^, ^17^ ^]^ However, in mammalian cells, Cas12j2 shows minimal editing efficiency, while Cas12j‐8 maintains measurable editing efficiency with high specificity.^[^ ^18^ ^]^ Despite this, Cas12j‐8 demonstrates suboptimal base editing capacity, and its potential for multiplex genome editing has yet to be fully explored.

This study utilized SWISS‐MODEL to predict the ternary structure of the Cas12j‐8/crRNA/dsDNA complex and applied a structure‐guided protein engineering strategy to systematically optimize Cas12j‐8, yielding an optimized variant, termed “engineered Cas12j‐8” (enCas12j‐8). The transcriptional activation tool developed from this engineered variant exhibited a 1.91‐ to 5.98‐fold enhancement in gene activation capacity compared to the wild‐type in mammalian cells. The engineered variant displayed a 1.75‐ to 55.09‐fold improvement in genome editing efficiency, a 2.43‐to 29.54‐fold increase in cytosine base editing activity, and a 4.21‐ to 36.57‐fold enhancement in adenine base editing performance relative to the wild‐type at the dominated sites. Notably, enCas12j‐8 enabled simultaneous editing of three genes using a single crRNA array. By overcoming the activity limitations of wild‐type Cas12j‐8 in mammalian systems, this study establishes a CRISPR‐Cas editing platform that integrates high efficiency, precision, and multiplex editing capability, providing a novel toolset for gene therapy of complex diseases.

## 2 Results

### 2.1 Engineering the Cas12j‐8 Protein for Enhanced Gene Activation in Mammalian Cells

**Figure 1:**
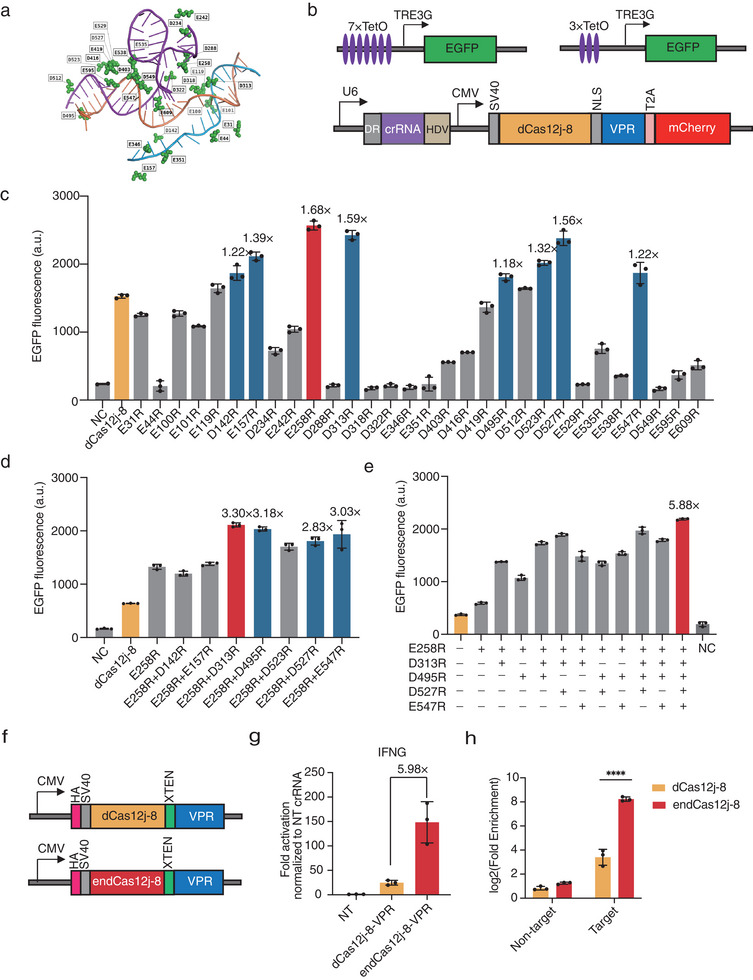
Optimized dCas12j‐8 Variants Enhance EGFP Reporter Activation and Endogenous Gene Expression a) Structural annotation of selected mutation sites in Cas12j‐8 homology modeling (PDB: 7LYS). The positions of individual point mutations tested are shown on the solved structure of Cas12j‐8 in complex with crRNA (blue), the target ssDNA (purple), and the non‐target ssDNA (orange). The locations of the focused amino acids are highlighted in green color with black letter labels. b) Schematic representation of the transfection constructs used for mutant screening. HEK293T cells stably expressing EGFP under the control of the inducible TRE3G promoter (top panel) were transfected with plasmids encoding wild‐type or mutant dCas12j‐8 and crRNA targeting TetO (bottom panel). EGFP expression was analyzed by flow cytometry 72 h post‐transfection. c) EGFP fluorescence levels in reporter cells transfected with wild‐type dCas12j‐8 or its single mutants. Yellow indicates wild‐type dCas12j8, red denotes the optimal mutant, blue represents suboptimal mutations selected as candidates for the next round of mutations. Data are shown as mean ± s.d. (n = 3 independent experiments except for negative control (NC) with two biological replicate experiments). a.u., arbitrary units. d) EGFP fluorescence levels in reporter cells transfected with wild‐type dCas12j‐8 and combinatorial variants containing several of the most potent single mutations in panel (c). Yellow indicates wild‐type dCas12j8, red denotes the optimal mutant, and blue represents suboptimal mutations selected as candidates for the next round of mutations. e) EGFP fluorescence levels in reporter cells transfected with wild‐type dCas12j‐8, the most potent single mutant, and combinatorial variants incorporating the strongest mutations identified in panel (d). Yellow indicates wild‐type dCas12j8, red denotes the optimal mutant. In panels (d, e), Data are shown as mean ± s.d., n = 3 independent experiments. a.u., arbitrary units. f) Schematic representation of constructs used for testing DNA binding affinity and gene activation efficiency at endogenous loci. g) Gene activation of *IFNG* using dCas12j‐8‐VPR and endCas12j‐8‐VPR, measured using RT‐qPCR in HEK293T cells. All data shown are fold activation of mRNA by normalizing to the mRNA expression using a non‐targeting crRNA (NT). Data are shown as mean ± s.d., n = 3 independent experiments. h) Comparative analysis of DNA binding affinity between dCas12j8 and endCas12j8. Fold enrichment of dCas12j‐8 and endCas12j‐8 at target DNA regions (*IFNG TTS*) quantified by ChIP‐qPCR. Data were normalized to input DNA and expressed as fold enrichment over non‐target control.  Fold enrichment values were log2‐transformed to visualize differential affinity. Data are shown as mean ± s.d. (n = 3 biological replicates). Differential binding between Cas12j8 and enCas12j8 was analyzed by two‐way ANOVA, **p < 0.01, ****p < 0.0001.

The Cas12j‐8 exhibits significantly weaker editing activity in mammalian cells compared to LbCas12a (Figure S1, Supporting Information), thereby limiting its potential applications in therapeutic gene editing. We hypothesize that protein engineering of Cas12j‐8 could improve this limitation. Structure‐guided protein mutagenesis is a highly cost‐effective strategy that has been validated in various experiments aimed at enhancing the activity of Cas proteins.^[^ ^19^, ^20^, ^21^ ^]^Although the structure of Cas12j‐8 has not yet been resolved, the cryo‐EM structure of the homologous protein Cas12j2 in complex with target DNA has been determined (PDB: 7LYS).^[^ ^22^ ^]^ Therefore, we utilized the protein structure prediction tool SWISS‐MODEL to generate a structural model of the Cas12j‐8 and nucleic acid complex.^[^ ^23^ ^]^ Our focus was on the negatively charged amino acids (aspartic acid and glutamic acid) within 8 Å of the target DNA and crRNA scaffold in Cas12j‐8 (**Figure** **1**a).^[^ ^12^, ^24^, ^25^, ^26^ ^]^ We systematically mutated these amino acid residues to positively charged arginine. We hypothesize that this modification can enhance the affinity between Cas12j‐8 and the negatively charged nucleic acids. To facilitate high‐throughput evaluation of different mutants, we constructed two HEK293T cell lines with genomic integration of TRE3G‐EGFP, integrating either 7×TetO or 3×TetO (tetracycline operator) using the piggyBac transposon system (Figure 1b). The CRISPR‐mediated activation (CRISPRa) was achieved by direct fusion mutated dCas12j‐8 (D369A, E567A, and D658A, a catalytically dead Cas12j‐8) to a tripartite VP64‐P65AD‐Rta (VPR) transcriptional activator.^[^ ^27^ ^]^And a 3′‐terminal self‐cleaving ribozyme HDV was inserted downstream of the TRE3G promoter‐targeting crRNA, which was reported can boosted the CRISPRa activity of the AsCas12a (Figure 1b).^[^ ^28^ ^]^ An all‐in‐one plasmid encoding the mutated dCas12j‐8‐VPR and crRNA was transfected into the reporter cells. The performance of variants was quantitatively compared using flow cytometry. In the 7×TetO reporter cell line, 8 out of 30 single‐point mutants exhibited more than a 1.1‐fold enhancement in transcriptional activation mediated by wild‐type dCas12j‐8. The most effective variant, E258R, achieved an increase of up to 1.68‐fold (Figure 1c).

For our second round of iteration, we introduced additional sites that were shown to enhance activity in the first round into the E258R variant, creating a library of seven double mutants. Using the 3×TetO reporter cells, we observed that E258R/D313R, E258R/D495R, E258R/D527R and E258R/E547R showed improvements over the E258R variant. Notably, E258R/D313R and E258R/D495R showed over a 3.1‐fold improvement in activation compared with wild‐type dCas12j‐8 (Figure 1d). For third round of iteration, we chose the single mutations (E258R, D313R, D495R, D527R, and E547R), which were combined into double mutants E258R/D313R and E258R/D495R to form triple, quadruple, and quintuple variants. Their ability to activate EGFP was compared in the 3×TetO reporter cells. We observed that the quintuple mutant (E258R/D313R/D495R/D527R/E547R) achieved the highest level of activation‐approximately 5.88‐fold above the level achieved by the wild‐type protein (Figure 1e). Hereafter, we refer to this quintuple mutant as “engineered dCas12j‐8” (endCas12j‐8).

We assessed the transcriptional activation capacity of endCas12j‐8‐VPR at endogenous loci by designing four crRNAs targeting regions within 300 bp of *IFNG* and *KLF4* transcription start sites (TSS). In HEK293T cells, endCas12j‐8‐VPR demonstrated 1.91‐fold and 5.98‐fold enhanced activation of *KLF4* and *IFNG* expression respectively compared to wild‐type counterparts (Figure 1g; Figure S2, Supporting Information).

To determine if improved activation stemmed from increased DNA binding affinity, we performed ChIP‐qPCR analysis using HA‐tagged dCas12j‐8‐VPR targeting the *IFNG* TSS (Figure 1f). The engineered variant showed significantly greater target site enrichment than wild‐type dCas12j‐8 (Figure 1h), supporting our hypothesis that substituting negatively charged residues with positively charged arginine enhances nucleic acid interactions.

### 2.2 Engineered Cas12j‐8 Enables Robust Genome Editing in Mammalian Cells

**Figure 2:**
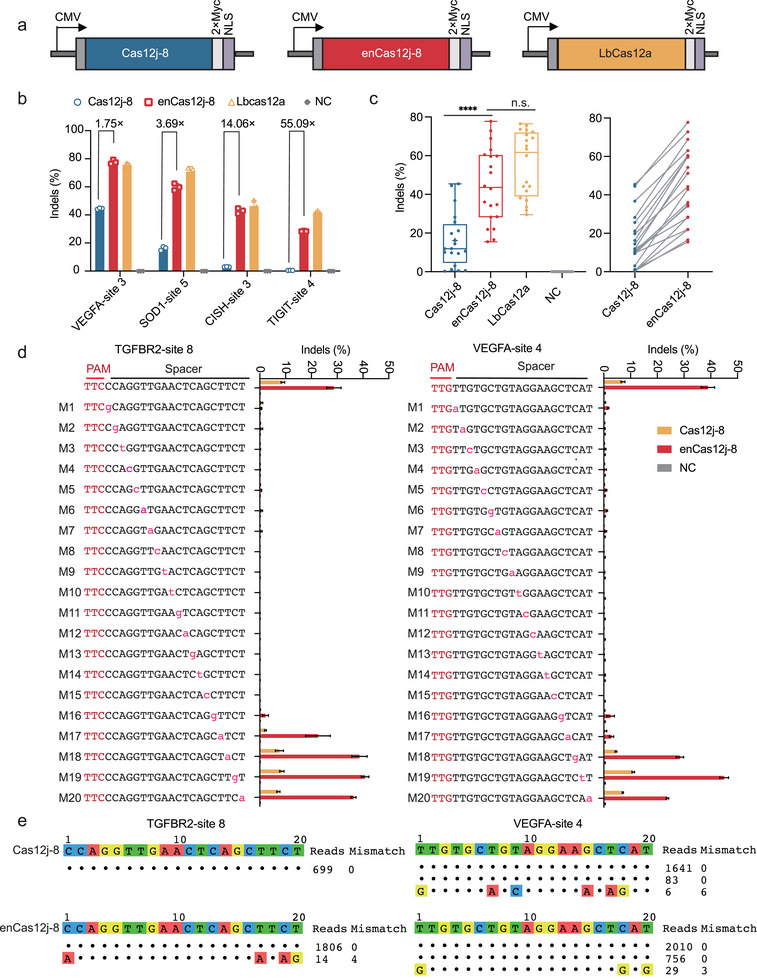
Specificity of Engineered Cas12j‐8‐Mediated Gene Editing in Mammalian Cells a) Schematic of constructs used for testing gene editing efficiency at endogenous loci. b) Comparison of gene editing efficiencies between Cas12j‐8, enCas12j‐8, and LbCas12a at endogenous genomic loci in HEK293T cells. The mean ± s.d. and individual data points for n = 3 independent biological replicates are shown. c) The box plot on the left shows the summary of editing efficiencies by Cas12j‐8, enCas12j‐8, and LbCas12a at 20 endogenous target sites in HEK293T cells, as shown in the experiment in (b) and Figure S4 (Supporting Information). Each data point represents the mean of three replicates. The center line shows medians of all data points, and the box limits correspond to the upper and lower quartiles, while the whiskers extend to the largest and smallest values. The line chart on the right shows per‐site, paired comparisons between the editing efficiencies of Cas12j‐8 and enCas12j‐8. Statistical significance was evaluated using one‐way ANOVA followed by Šidák's multiple comparisons test. n.s., not significant; *p < 0.05; **p < 0.01; ***p < 0.001; ****p < 0.0001. d) Off‐target analysis of Cas12j‐8 and enCas12j‐8 at VEGFA and TGFBR2 with single mismatched protospacers using high‐throughput sequencing. Base mismatches are labelled in Red. The mean ± s.d. and individual data points are shown for n = 3 independent biological replicates. e) The genome‐wide off‐target effects of Cas12j‐8 and enCas12j‐8 are analyzed by GUIDE‐seq. On‐target and off‐target sequences are shown on the left. Read numbers are shown on the right. Mismatches compared to the on‐target site are shown and highlighted in color.

To investigate whether engineered substitutions enhance Cas12j‐8′s DNA cleavage efficiency, we assessed nuclease‐active variants at two endogenous loci. High‐throughput sequencing (HTS) showed progressively increased indel formation with incorporation of beneficial mutations. The quintuple mutant enCas12j‐8 (E258R/D313R/D495R/D527R/E547R) demonstrated peak editing efficiency at VEGFA and SOD1 loci in HEK293T cells (Figure S3, Supporting Information), indicating that rationally enhanced DNA binding affinity correlates with improved catalytic activity. We subsequently compared enCas12j‐8′s performance against wild‐type Cas12j‐8 and LbCas12a at four genomic targets: VEGFA, SOD1, CISH3, and TIGIT. We observed that enCas12j‐8 was comparable to LbCas12a at VEGFA (77.75% versus 75.64%) and CISH3 (42.91% versus 46.32%) (**Figure** **2**b). To further characterize gene editing using enCas12j‐8, we performed site‐by‐side comparison of enCas12j‐8, wild‐type Cas12j‐8, and LbCas12a at an additional 16 genomic sites (Figure 2c; Figure S4, Supporting Information). We observed more robust gene editing using enCas12j‐8 at these sites than the wild‐type.

### 2.3 Engineered Cas12j‐8 Retains High Editing Specificity

To comprehensively evaluate the editing specificity of enCas12j‐8, we assessed its tolerance to single‐nucleotide mismatches across the spacer region. Systematic single‐base mismatches were introduced into crRNAs targeting VEGFA‐site 4 and TGFBR2‐site 8, and editing efficiencies were measured by HTS. We observed that enCas12j‐8 exhibited high sensitivity to mismatches at PAM‐proximal positions (1‐15), with indel frequencies reduced to background levels in this region (Figure 2d). By contrast, moderate editing activity was retained when mismatches were introduced at PAM‐distal positions (16‐20), indicating relatively higher tolerance to mismatches in the distal region. These results demonstrate that enCas12j‐8 maintains stringent sequence specificity, particularly near the PAM, and discriminates against mismatches in a position‐dependent manner.

Next, we performed GUIDE‐seq^[^ ^29^ ^]^ to analyze genome‐wide off‐target effects of Cas12j‐8 and enCas12j‐8 in HEK293T cells. At the TGFBR2‐site8 locus, no off‐target sites were detected for Cas12j‐8. Notably, enCas12j‐8 exhibited only one off‐target site with extremely low editing efficiency despite its enhanced activity. At the VEGFA‐site4 locus, both Cas12j‐8 and enCas12j‐8 showed a single off‐target site with similarly low editing efficiency (Figure 2e). These findings align with previous reports confirming Cas12j‐8′s low off‐target propensity, while demonstrating that enCas12j‐8 maintains minimal off‐target effects even with significantly improved on‐target editing activity, achieving precision‐enhanced genome editing.

### 2.4 Engineered Cas12j‐8 Exhibits Broad Activity Across Mammalian Cell Types

To evaluate the generalizability of enCas12j‐8 across different mammalian cell types, we assessed its genome editing activity in three additional human cell lines: HCT116, HeLa, and A549, in addition to HEK293T cells. Across all tested cell lines, enCas12j‐8 demonstrated significantly higher editing efficiencies compared to the wild‐type Cas12j‐8. Notably, the enhancement was observed at multiple target sites within each cell type, indicating that the improved performance of enCas12j‐8 is not restricted to a specific genomic context or cellular background (Figure S5, Supporting Information). These results confirm that the rationally engineered variant maintains robust and broadly applicable editing activity, supporting its potential for applications in diverse biological systems.

### 2.5 Cytosine Base Editors Using Engineered dCas12j‐8

**Figure 3:**
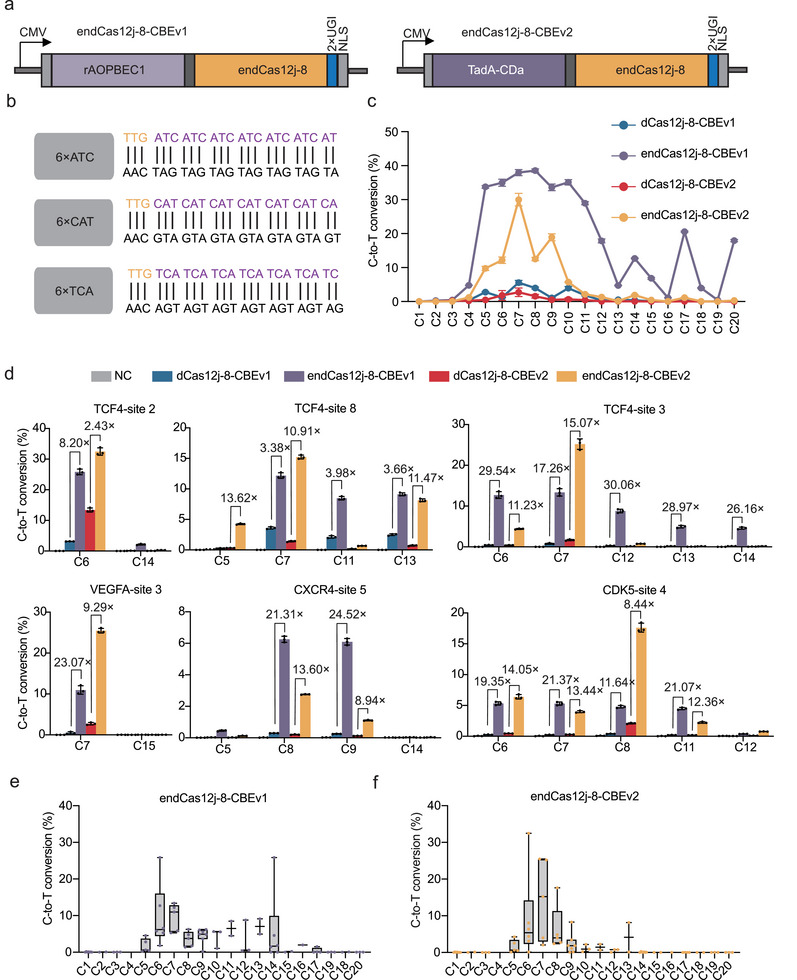
Editing Efficiency of Engineered dCas12j‐8‐Mediated Cytosine Base Editing a) Schematic of two versions of cytosine base editors (CBEs) based on endCas12j‐8. b) Diagram of repetitive sequences used to assess the base editing window. By embedding 6×ATC,6×CAT, and 6×TCA (purple) into the spacer region downstream of the PAM sequence (TTG, yellow) of Cas12j‐8, three plasmids containing repeat sequences were constructed to cover all spacer position (1 to 20 nt) that may be edited by ABE/CBE of dCas12j‐8 and endCas12j‐8. c) Line graph depicting the C‐to‐T conversion (%) by CBEv1 and CBEv2 at each protospacer position (1 to 20 nt). Data represent mean ± s.d., with individual data points from three independent experiments (n = 3) in HEK293T cells. d) Evaluation of the C‐to‐T conversion (%) of CBEv1 and CBEv2 at six representative endogenous genomic loci. Data represent mean ± s.d., with individual data points from three independent experiments (n = 3) in HEK293T cells. e) The box plot shows the summary of C‐to‐T conversion (%) by endCas12j‐8‐CBEv1 at 15 endogenous target sites in HEK293T cells, as shown in (d) and Figure S6 (Supporting Information). f) The box plot shows the summary of C‐to‐T conversion (%) by endCas12j‐8‐CBEv2 at 15 endogenous target sites in HEK293T cells, as shown in (d) and Figure S6 (Supporting Information). In panels e and f, the center line shows medians of all data points, and the box limits correspond to the upper and lower quartiles, while the whiskers extend to the largest and smallest values.

Previous studies applied Cas12j‐8 to base editing, revealing that it exhibited very low activity in adenine base editing (ABE) at only a limited number of sites, with no detectable cytosine base editing (CBE) activity at any site in human cells.^[^ ^18^ ^]^ We hypothesized that endCas12j‐8 could overcome these limitations. Therefore, we created two versions of CBEs based on endCas12j‐8. The first version (endCas12j‐8‐CBEv1) was designed by fusing the cytidine deaminase rAPOBEC1 with two uracil glycosylase inhibitor domains (2×UGI).^[^ ^30^, ^31^ ^]^ The second version (endCas12j‐8‐CBEv2) utilized an evolved TadA‐derived cytidine deaminase (TadA‐CDa)^[^ ^32^ ^]^ instead of rAPOBEC1 (**Figure** **3**a). Using the same approach, two corresponding CBEs were also generated with dCas12j‐8 (dCas12j‐8‐CBEv1 and dCas12j‐8‐CBEv2). To characterize the editing efficiency and window of these base editors, we constructed spacer plasmids containing 6×ATC, 6×CAT, and 6×TCA repeats downstream of the TTG sequence (Figure 3b). Upon co‐transfecting cells with different dCas12j‐8‐derived CBEs and the crRNA plasmids targeting specific positions within the spacer, HTS results demonstrated that endCas12j‐8‐CBEv1 achieved an editing efficiency of up to 38.57% at the C8 position, exhibiting a broad editing window (primarily between C4‐C13, with partial effectiveness at C14‐C15, C17‐C18, and C20) compared to other CBE variants. In contrast, endCas12j‐8‐CBEv2 showed a narrower editing window (C5‐C10), with a peak editing efficiency of 29.99% at the C7 position (Figure 3c). We then explored the potential of these CBEs at endogenous loci in human cells (Figure 3d). In the representative endogenous sites, endCas12j‐8‐CBEv1 exhibited notable editing efficiencies. Specifically, at the TCF4 site 3, TCF4 site 8, and VEGFA site 3 (C7 position) the editing efficiencies were 13.37%, 12.18%, and 10.97%, representing 17.26‐fold, 3.38‐fold, and 23.07‐fold increases compared to the wild‐type, respectively. Similarly, endCas12j‐8‐CBEv2 achieved editing efficiencies of 25.20%, 15.23%, and 25.48% at the same sites, corresponding to 15.07‐fold, 10.91‐fold, and 9.29‐fold enhancements over the wild‐type. For CXCR4 site 5 and CDK5 site 4 (C8 position), endCas12j‐8‐CBEv1 demonstrated editing efficiencies of 6.26% and 4.78%, with respective increases of 21.31‐fold and 11.64‐fold relative to the wild‐type. Likewise, endCas12j‐8‐CBEv2 exhibited editing efficiencies of 2.76% and 17.62% at these sites, corresponding to 13.60‐fold and 8.44‐fold enhancements over the wild‐type. These results indicate that endCas12j‐8 significantly enhances the editing efficiency of C‐to‐T conversions (Figure 3d).

To further characterize the editing properties of endCas12j‐8‐CBEs, we analyzed the editing efficiencies at additional endogenous sites (Figure S6, Supporting Information). Interestingly, we found that endCas12j‐8‐CBEv2 exhibited dominated editing activity at the C7 position and had a narrower editing window compared to the v1 version (Figure 3e,f).

### 2.6 Adenine Base Editors Using Engineered dCas12j‐8

**Figure 4:**
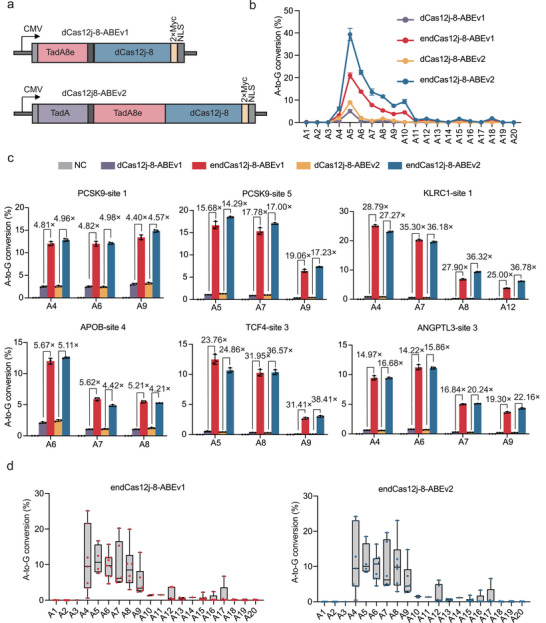
Editing Efficiency of Engineered dCas12j‐8‐Mediated Adenine Base Editing a) Schematic representation of the constructs used for two versions of dCas12j‐8 adenine base editors (ABE). b) Line graph illustrating the A‐to‐G conversion (%) by ABEv1 and ABEv2 at each protospacer position (1 to 20 nt). Data represent mean ± s.d., with individual data points from three independent experiments (n = 3) in HEK293T cells. c) Evaluation of the A‐to‐G conversion (%) of ABEv1 and ABEv2 at six representative endogenous genomic loci. Data represent mean ± s.d., with individual data points from three independent experiments (n = 3) in HEK293T cells. d) The box plot shows the summary of A‐to‐G conversion (%) by endCas12j‐8‐ABEv1 (left) and endCas12j‐8‐ABEv2 (right) at 14 endogenous target sites in HEK293T cells, as shown in c and Figure S7 (Supporting Information). The center line shows medians of all data points, and the box limits correspond to the upper and lower quartiles, while the whiskers extend to the largest and smallest values.

Next, adenine base editors (ABEs) were generated by fusing dCas12j‐8 variants to adenine deaminases. We developed two versions of endCas12j‐8‐based adenine base editors: endCas12j‐8‐ABEv1, which consists of endCas12j‐8 fused to the engineered adenine deaminase TadA (TadA8e),^[^ ^33^ ^]^ and endCas12j‐8‐ABEv2, which combines endCas12j‐8 with a dimer of TadA, including both wild‐type tRNA‐specific adenosine deaminase (wtTadA) and TadA8e, which has been reported to exhibit higher A‐to‐G conversion efficiency compared to TadA8e in human cells (**Figure** **4**a).^[^ ^21^, ^34^ ^]^

We then evaluated the editing window of these ABEs using plasmids containing 6×ATC repeats (Figure 3b). The results revealed that both editors exhibited editing activity primarily within positions A4‐A10, with peak efficiency at A5, where endCas12j‐8‐ABEv1 achieved 20.97% and endCas12j‐8‐ABEv2 reached 39.34%. These findings demonstrate that these ABE variants incorporating endCas12j‐8 exhibit significantly higher adenine base editing efficiency than those incorporating wild‐type dCas12j‐8. (Figure 4b).

We subsequently evaluated their editing efficiency at endogenous loci. At PCSK9‐site 5 A5, endCas12j‐8‐ABEv1 exhibited a 15.68‐fold improvement in editing efficiency compared to dCas12j‐8‐ABEv1 (1.06% versus 16.67%). Similarly, endCas12j‐8‐ABEv2 exhibited a 14.29‐fold increase relative to dCas12j‐8‐ABEv2 (1.29% versus 18.49%).

At KLRC1‐site1 A4, the editing efficiency of endCas12j‐8‐ABEv1 reached 25.12%, representing a 28.79‐fold increase compared to dCas12j‐8‐ABEv1(Figure 4c).

To further validate these findings, we examined the efficiency of endCas12j‐8‐ABE editing at additional endogenous sites (Figure S7, Supporting Information). Together, these results indicate that endCas12j‐8‐based ABEs achieve substantial improvements in base editing efficiency (Figure 4d; Figure S7, Supporting Information).

**Figure 5:**
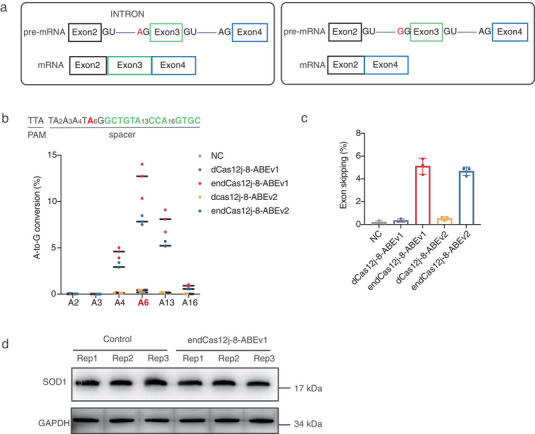
dCas12j‐8‐ABE was used for single‐base editing of the spliceosome consensus sequence to achieve programmable exon skipping. a) Schematic representation of single‐base editing of spliceosome consensus sequences to achieve programmable exon skipping. The left panel shows the normal splicing process of SOD1 mRNA, and the right panel shows the process of exon skipping when the splice acceptor sequence is disrupted. b) Percentage of sequencing reads showing A‐to‐G conversion (%) in HEK293T genome under various dCas12j‐8‐ABE conditions targeting the splice acceptor (red A) of Exon 3. c) Percentage of sequencing reads showing SOD1 Exon3 skipping (%) in HEK293T cDNA under various dCas12j‐8‐ABE conditions targeting the splice acceptor (red A) of Exon 3. In panels (c, d), data represent mean ± s.d., with individual data points from three independent experiments (n = 3) in HEK293T cells. d)Western blot analysis of SOD1 protein levels following exon skipping in HEK293T cells, GAPDH was used as a loading control.

Pre‐mRNA splicing removes introns through two transesterification reactions occurring at conserved 5′ splice site (GU), 3′ splice site (AG) splice sites (GU‐AG rule), and a branch point. Disrupting the 3′ splice site via adenine‐to‐guanine (A‐to‐G) conversion can induce exon skipping (**Figure** **5**a), a strategy that may reduce the expression of pathogenic proteins.^[^ ^35^ ^]^ We engineered endCas12j‐8‐ABE editors to target the SOD1 (associated with Amyotrophic Lateral Sclerosis, ALS)^[^ ^36^ ^]^ exon 3 3′ splice site using a TTA PAM‐specific crRNA. In HEK293T cells, ABEv1 achieved 12.37% A‐to‐G editing and 5.11% exon skipping (Figure 5b,c), demonstrating proof‐of‐concept for splice‐site‐targeted exon skipping. While our RNA‐level data confirmed successful exon skipping at the SOD1 locus, the limited editing efficiency resulted in non‐significant alterations in SOD1 protein levels (Figure 5d), further optimization is required to enhance editing efficiency and assess functional outcomes for therapeutic applications.

### 2.7 Multiplexed Gene and Base Editing with Engineered Cas12j‐8

**Figure 6:**
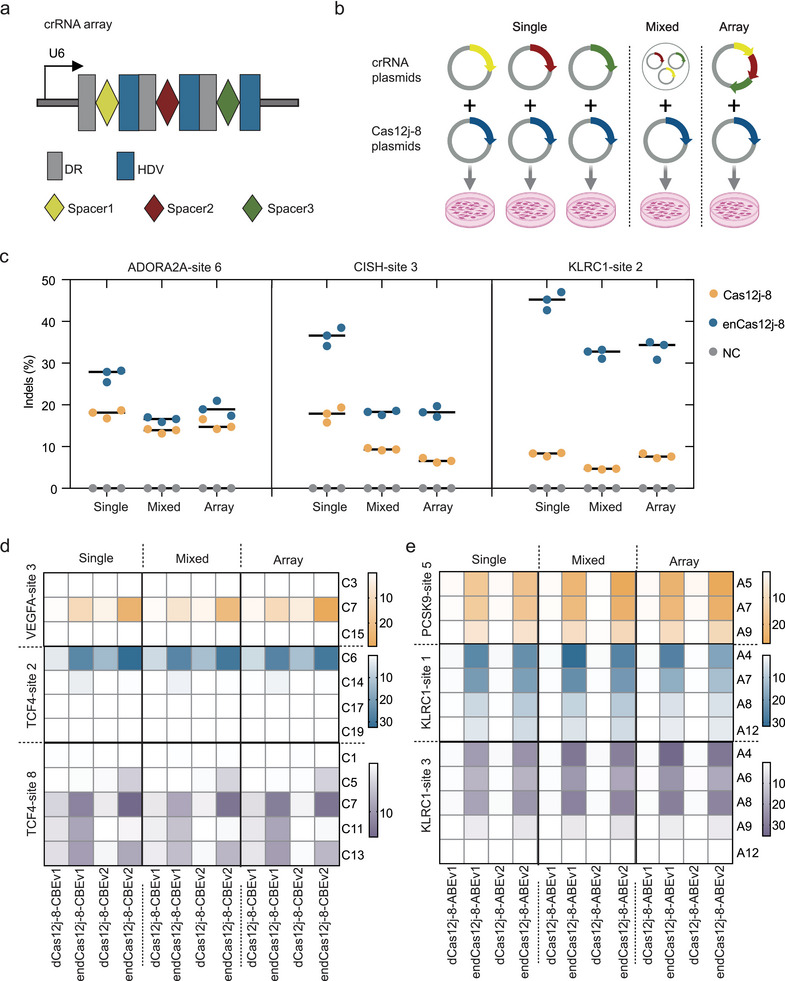
Engineered Cas12j‐8‐Mediated Multiplexed Gene Editing in Mammalian Cells a) Schematic of constructs used for multiplexed gene editing, featuring a CRISPR‐crRNA array with three crRNAs (different colored diamonds) separated by direct repeats (DR). b) Schematic representation of the transfection strategy used to detect Cas12j‐8 multiplexed gene editing in the HEK293T. Different crRNA spacers are indicated in different colors. Created in BioRender. SAN, L. (2025) https://BioRender.com/r99y984. (Agreement number: UD27WE5UGG) c) Editing efficiencies of Cas12j‐8 and enCas12j‐8 at three endogenous genomic loci simultaneously using a crRNA array in HEK293T cells, compared to single crRNA editing and mixed‐crRNA editing. Data represent the mean of three independent experiments. d) C‐to‐T conversion (%) of CBEv1 and CBEv2 at three endogenous genomic loci simultaneously using a crRNA array in HEK293T cells, compared to single crRNA editing and mixed‐crRNA editing. Data represent the mean of three independent experiments. e) A‐to‐G conversion (%) of ABEv1 and ABEv2 at three endogenous genomic loci simultaneously using a crRNA array in HEK293T cells, compared to single crRNA editing and mixed‐crRNA editing. Data represent the mean of three independent experiments.

Cas12j uses a single RuvC active site for both pre‐crRNA maturation and DNA cleavage, suggesting its capability to catalyze crRNA maturation independently.^[^ ^13^ ^]^ This feature enables Cas12j to potentially utilize a single CRISPR array for multiplexed editing. To evaluate the multiplexed gene and base editing capabilities of enCas12j‐8, we employed a crRNA array containing three distinct spacer sequences targeting different genomic loci (**Figure** **6**a) and compared its editing efficiency with that achieved by transfecting these three single crRNAs separately or mixed (Figure 6b). We measured indel formation efficiency using HTS at three genomic sites (ADORA2A‐site 6, CISH3‐site 3, and KLRC1‐site 2). We found that enCas12j‐8 could efficiently edit multiple gene targets simultaneously, achieving significantly higher editing efficiency than wild‐type Cas12j‐8 (Figure 6c). Although the editing efficiency of the crRNA array was slightly lower than that achieved by single‐crRNA editing alone, it remained comparable to the efficiency observed in mixed‐crRNA editing (Figure 6c), confirming its applicability for multiplexed gene editing.

We next assessed the multiplexed editing capabilities of the cytosine base editor (CBE). To do this, a crRNA array was constructed targeting VEGFA‐site 3, TCF4‐site 1, and TCF4‐site 2 (Figure 6d). The results indicated that the editing efficiency of multiplexed editing was comparable to single‐crRNA editing. At VEGFA‐site 3 C7, the maximum editing efficiencies achieved with single‐crRNA, mixed‐crRNA, and crRNA array were 25.48%, 22.17%, and 29.23%, respectively, using endCas12j‐8‐CBEv2. Similarly, at TCF4‐site 1 C6, the efficiencies were 32.48%, 29.75%, and 29.48%, while at TCF4‐site 2 C7, they were 15.23%, 13.93%, and 13.95%, respectively, using endCas12j‐8‐CBEv2. (Figure 6d). These results indicate that the crRNA array achieves editing efficiencies comparable to, if not slightly better than, the co‐transfection of multiple crRNAs.

To evaluate the ABEs multiplexed editing capabilities of endCas12j‐8, we constructed a crRNA array targeting PCSK9‐site 5, KLRC1‐site 1, and KLRC1‐site 2 (Figure 6e). Notably, the editing efficiency of the crRNA array exceeded that of single‐crRNA editing. The maximum editing efficiencies achieved at PCSK9‐site 5 A5 with single‐crRNA, mixed‐crRNA, and crRNA array were 18.49%, 26.84%, and 27.13% by endCas12j‐8‐ABEv2, respectively, while at KLRC1‐site 1 A4, the efficiencies were 25.12%, 31.76%, and 26.57% by endCas12j‐8‐ABEv1, and at KLRC1‐site 2 A4, they were 24.19%, 29.28%, and 31.6% by endCas12j‐8‐ABEv2 (Figure 6e). In summary, these findings indicate that enCas12j‐8 can efficiently perform multiplexed editing at multiple genomic loci, underscoring its potential as a versatile tool for genome engineering.

## 3 Discussion

The engineering of Cas12j‐8 through structure‐guided mutagenesis has significantly enhanced its utility as a versatile genome‐editing tool in mammalian cells. By systematically replacing negatively charged residues near the DNA‐binding interface with positively charged arginine, we generated an engineered variant, enCas12j‐8, which demonstrated a remarkable over fivefold improvement in transcriptional activation compared to the wild‐type protein. This improvement was experimentally validated through ChIP‐qPCR, which confirmed that enCas12j‐8 has significantly higher DNA‐binding affinity at target loci. These findings demonstrate that rational charge‐reversal mutagenesis can effectively enhance protein–nucleic acid interactions and provide a broadly applicable strategy for CRISPR optimization. Beyond transcriptional regulation, we showed that enhanced DNA binding also translated into improved genome editing efficiency. Notably, enCas12j‐8 also exhibited robust gene‐editing efficiency comparable to LbCas12a at multiple genomic loci while maintaining low off‐target activity, underscoring its potential for therapeutic applications requiring high precision. This increase in on‐target activity was consistent across multiple human cell lines, including HCT116, HeLa, and A549 cells, supporting its general applicability in various cellular contexts.

The study suggests that in the absence of the actual structure of Cas proteins, the protein‐nucleic acid complex structures predicted by the SWISS‐MODEL tool, which is based on homologous protein modeling, can also be used for structure‐based rational protein design. Recent researchers have used AlphaFold2 to optimize Cas12j‐8, demonstrating that the A121Q/S196Q double mutant can improve the editing efficiency of Cas12j‐8 in plant systems such as soybean and rice.^[^ ^17^ ^]^ This indicates that introducing other sites and mutation types may further enhance the activity of enCas12j‐8.

The development of cytosine and adenine base editors (CBEs and ABEs) based on enCas12j‐8 addressed the previously reported limitations of Cas12j‐8 in base editing.^[^ ^18^ ^]^ The enCas12j‐8‐CBEv1 and enCas12j‐8‐CBEv2 variants achieved broad and narrow editing windows, respectively, with substantial improvements in C‐to‐T conversion efficiency. Similarly, enCas12j‐8‐ABEv2, incorporating a dimeric TadA system, showed near 40% editing efficiency at adenine residues, outperforming wild‐type dCas12j‐8‐based editors. These advancements highlight the adaptability of enCas12j‐8 for diverse editing modalities and its compatibility with deaminase engineering strategies. Future efforts could focus on enhancing base editing specificity by engineering deaminase domains, exploring alternative effector fusions, and optimizing crRNA design, with the goal of narrowing the editing window and minimizing bystander edits.

Furthermore, the application of enCas12j‐8‐ABE for CRISPR‐SKIP‐mediated exon skipping in SOD1 exemplifies its therapeutic potential, albeit with room for optimization to achieve clinically relevant efficiency. While our RNA‐level data confirmed successful exon skipping at the SOD1 locus, the limited editing efficiency resulted in non‐significant alterations in SOD1 protein levels. To establish stronger therapeutic evidence, systematic evaluation of exon skipping's functional impact in ALS mouse model will be prioritized in our subsequent investigations.

A key innovation of this study lies in the demonstration of multiplexed editing using enCas12j‐8. By leveraging its intrinsic crRNA‐processing capability, enCas12j‐8 efficiently edited multiple genomic targets simultaneously via a single CRISPR array. This feature is particularly advantageous for complex genome engineering tasks, such as combinatorial gene regulation or correcting polygenic disorders. Despite the RuvC domain being proven necessary for pre‐crRNA processing in vitro,^[^ ^13^ ^]^ we were initially surprised to find that in HEK293T cells, RuvC‐inactive endCas12j‐8‐mediated CBE and ABE were still capable of multi‐target editing. We hypothesized that the 3′‐terminal HDV ribozyme, previously reported to enhance crRNA maturation,^[^ ^17^, ^37^ ^]^ might compensate for the loss of RuvC activity. However, follow‐up experiments revealed that efficient multiplex editing persisted even when the HDV ribozyme was removed from the CRISPR array construct (Figure S8, Supporting Information). These results suggest that HDV is not essential for crRNA maturation in this context, and that endCas12j‐8 retains intrinsic crRNA processing capability in mammalian cells. The exact mechanism remains to be fully elucidated, but our findings highlight the robustness and flexibility of enCas12j‐8 in multiplexed genome editing applications.

For therapeutic translation, achieving precise and tissue‐restricted genome editing is critical to minimize systemic off‐target effects. Although tissue‐specific delivery strategies were not explored in this study, future work may incorporate cell type‐specific or inducible promoters, viral vectors with tailored tissue tropism, or localized delivery approaches such as intrathecal or intramuscular injection. Integration of such strategies with enCas12j‐8 could enable safe and efficient in vivo genome modulation for therapeutic purposes.

In conclusion, enCas12j‐8 represents a significant advancement in the CRISPR toolbox, combining high efficiency, precision, and multiplexing capabilities. Its success in base editing, transcriptional activation, and exon skipping underscores its broad applicability in both basic research and therapeutic development.

## 4 Experimental Section

### Cell Culture and Transfection

HEK293T cells (ATCC) and Hela (ATCC) were cultured in DMEM (Thermo Fisher). HCT116 (ATCC) cells were maintained in McCoy's 5A Medium (Thermo Fisher), and A549 (ATCC) cells were grown in RPMI‐1640 Medium (Thermo Fisher). All media were supplemented with 10% fetal bovine serum (FBS, Vazyme) and 1% penicillin–streptomycin (Thermo Fisher). The cells were maintained at 37 °C and 5% CO2. To ensure optimal proliferation, culture medium for HCT116 and A549 cells was refreshed every 48 h. Cells were passaged at 80 – 90% confluence using 0.25% Trypsin‐EDTA (Thermo Fisher). For transfection of cells, the cells were seeded in 12‐well plates the day before transfection and transfected when the cell density reached 70%‐80%. Transfection was performed using 2 µL of transfection reagent (Vazyme) per µg of plasmid DNA. The reagents and plasmids were diluted separately with 100 µL of Opti‐MEM (Thermo Fisher), thoroughly mixed, and incubated for 15 min before being added to the cells. After 24 h of transfection, the cells were treated with 40 µg mL^−1^ Blasticidin (InvivoGen) and 4 µg mL^−1^ puromycin (InvivoGen) for 24 h. For cells used for flow analysis, samples were collected 72 h after transfection. For high‐throughput sequencing of the extracted genome, cells were resuspended 72 h after transfection, with samples collected at 96 h.

### Plasmid Cloning

Plasmids were cloned using standard molecular cloning techniques, including PCR amplification, restriction enzyme digestion, and ligation. All Cas constructs were constructed with pST1374 vector. The amino acid sequences of the Cas proteins used in this study are shown in Table S1 (Supporting Information). Amplification of all Cas constructs was performed using Phanta Max Super‐Fidelity DNA Polymerase (Vazyme). Following digestion with DpnI (New England BioLabs), the PCR products were ligated using 2×MultiF Seamless Assembly Mix (ABclonal). Ligated products were transformed into DH5α E. coli cells. The PGL3‐U6‐crRNA vector was constructed as reported previously using the PGL3 backbone. All crRNA plasmids were cloned by annealing oligos for targeting spacers and ligating them into BsaI‐HFv2 (New England BioLabs) digested backbone vectors using T4 DNA Ligase (New England BioLabs). Assembly of CRISPR arrays was performed using Golden Gate Cloning via BsmBI restriction enzyme to insert either single or multiple spacers, using annealed oligonucleotide (Sangon Biotech). The sequences of the plasmids are shown in Table S2 (Supporting Information). The spacer sequences of crRNAs used in the study are shown in Table S3 (Supporting Information). The final constructed vectors were all validated for accuracy through Sanger sequencing.

### Flow Cytometry

Cells were dissociated using 0.25% trypsin‐EDTA (Thermo Fisher), resuspended in DMEM, and analyzed for fluorescence intensity using a BD FACSVerse flow cytometer. Cells (10000) from the gated population of interest were collected from each sample. Mean fluorescence intensity was analyzed using FlowJo (v10).

### High‐throughput Sequencing and Data Analysis

High‐throughput sequencing (HTS) primers were designed to generate amplicons of 200–270 bp encompassing the endogenous locus, incorporating specific adapters, as shown in Table S3 (Supporting Information). For the next‐generation sequencing experiment, cells were washed with PBS (Gibco) and subjected to genomic DNA extraction using QuickExtract DNA Extraction Solution (Lucigen) 96 hours post‐transfection. The cells in QuickExtract solution were incubated at 65 °C for 60 min and then heat inactivated at 98 °C for 2 min. Phanta Max Super‐Fidelity DNA Polymerase (Vazyme) was used to amplify the target sequence with the following cycle conditions: 98 °C for 3 min, [98 °C for 15 s, 60 °C for 15 s, 72 °C for 30 s]x29 cycles, 72 °C for 3 min. Quantitative analysis was performed on the PCR products using ImageJ. The products with different barcode were pooled in equimolar amounts. Concentration was determined using the Equalbit dsDNA HS Assay Kit (Vazyme) after purification. Then, 10 ng of the product from each pool was used for amplification with index primers (VAHTS Universal DNA Library Prep Kit for Illumina V4, Vazyme) using 2×HIFI (Roche). The samples were pooled in equimolar amounts, and a mixed barcoded library was sequenced on the Illumina NovaSeq platform. Data analysis was performed using CRISPResso2 (https://github.com/pinellolab/CRISPResso2).

### Extraction of Total RNA and Quantitative Real‐Time PCR (qRT‐PCR)

RNA was isolated from 6 days transfected cells using RNAiso Plus (TaKaRa). Total RNA was assessed for purity and concentration using NanoDrop One. Then 500 ng of RNA was used for reverse transcription with the HiScript III RT SuperMix for qPCR (+gDNA wiper) (Vazyme), following the reagent instructions, and the total volume was diluted to 100 µL with Nuclease‐Free Water (Thermo Fisher). qPCR was performed using the QuantStudio 7 Flex Real‐Time PCR System and the SupRealQ Purple Universal SYBR qPCR Master Mix (U+) (Vazyme). The primer sequences are shown in Table S3 (Supporting Information).

### Chromatin Immunoprecipitation (ChIP) and qPCR Analysis

Transfected HEK293T cells in 10‐cm Petri dishes were cross‐linked with 1% formaldehyde (Sigma) for 10 min at room temperature to stabilize protein–DNA interactions. The reaction was quenched with 125 mM glycine (Sangon biotech) for 5 min at room temperature. Cells were then washed twice with ice‐cold PBS containing protease inhibitors (Selleckchem). Cross‐linked cells were resuspended in SDS lysis buffer (50 mM Tris‐HCl, pH 8.0; 10 mM EDTA; 1% SDS) containing protease inhibitors. Chromatin was fragmented using a Covaris S220 sonicator (Peak Power: 105 W, Duty Factor: 5%, Cycles: 200, Treatment Time: 40 s) to yield DNA fragments of 200–1000 bp. Fragment size was verified by 1.5% agarose gel electrophoresis. The lysates were centrifuged at 5000 × *g* for 10 min at 4 °C to remove insoluble material.

The chromatin lysate was diluted by adding 900 µL of ChIP dilution buffer (10 mM Tris‐HCl, pH 7.5; 150 mM NaCl; 0.1% NP‐40) and 20 µL of protease inhibitors (Sigma) to 100 µL of supernatant. ChIP‐grade Protein A/G magnetic beads (Thermo Fisher) were added and incubated at 4 °C for 2 h. After removing the beads, the supernatant was retained. A 20 µL aliquot of each sample was saved as input. One immunoprecipitation tube was incubated with 1 µL of HA antibody (Proteintech), while a control tube was left without antibody. Both were incubated overnight at 4 °C.

Beads were sequentially washed with low‐salt buffer (20 mM pH 8.0 Tris‐HCl, 150 mM NaCl, 2 mM EDTA, 1% NP‐40, 0.1% SDS), high‐salt buffer (20 mM pH 8.0 Tris‐HCl, 500 mM NaCl, 2 mM EDTA, 1% NP‐40, 0.1% SDS), LiCl buffer (10 mM pH 8.0 Tris‐HCl, 250 mM LiCl, 1 mM EDTA, 1% NP‐40; 1% sodium deoxycholate), and TE buffer (10 mM pH 8.0 Tris‐HCl, 1 mM EDTA). DNA–protein complexes were eluted in elution buffer (1% SDS, 100 mM NaHCO₃) for 15 min at room temperature for twice. Crosslinks were reversed by adding 200 mM NaCl and incubating at 65 °C overnight. Samples were then treated with RNase A (37 °C, 1 h) and 10 mM EDTA, 40 mM Tris.HCl, 0.05 mg mL^−1^ Proteinase K (45 °C, 2 h), followed by purification using phenol‐chloroform extraction.

Quantitative PCR was performed in triplicate using SupRealQ Purple Universal SYBR qPCR Master Mix (U+) (Vazyme) on the QuantStudio 7 Flex Real‐Time PCR System. Cycling conditions were: 95 °C for 5 min, followed by 40 cycles of 95 °C for 15 s and 60 °C for 30 s. Melt curve analysis was used to confirm primer specificity. Primer sequences are listed in Table S3 (Supporting Information). To quantify Cas protein‐DNA binding affinity, qPCR data were analyzed via the ΔΔCt method. Fold‐enrichment over the non‐target control. Data from three independent biological replicates were analyzed by unpaired Student's t‐test (****p < 0.001, **p < 0.01).

### GUIDE‐Seq

HEK293T cells (2 × 10⁵ cells per well) were transfected via electroporation with 1 µg Cas12j‐8 plasmid, 500 ng crRNA plasmid, and 10 pmol of annealed GUIDE‐seq oligonucleotides (5′‐TAATCGATACCGTTATTAACATATGACAACTCAATTAAACTCTGAA‐3′), followed by culture in 12‐well plates for 72 h. Genomic DNA was extracted, and target regions were amplified by PCR, purified via gel electrophoresis, and analyzed by Sanger sequencing (indel quantification) and restriction enzyme digestion coupled with capillary electrophoresis (cleavage efficiency). For HTS, ≥200 ng of genomic DNA was enzymatically fragmented, end‐repaired (3′ A‐tailing), and ligated to Illumina adaptors. Libraries underwent three rounds of PCR amplification (P5/P7 primers) with intermediate magnetic bead purification to remove primer dimers. Final libraries were quantified, quality‐checked, and sequenced on an Illumina HiSeq/Novaseq or MGI2000 platform (PE150 mode). Raw reads were demultiplexed and filtered (Cutadapt v1.18), followed by UMI‐based error correction (custom scripts), alignment to the hg38 genome (BWA v0.7.12‐r1039), and off‐target site identification^[^ ^29^ ^]^ (GUIDE‐seq pipeline). Gene annotations for on‐target and off‐target loci were performed using ANNOVAR (v2020‐06‐08, https://annovar.openbioinformatics.org/). All experiments included triplicate replicates, with off‐target sites defined by ≥ 3 overlapping GUIDE‐seq tags, ≥ 90% alignment score, and absence in negative controls.

### Statistical Analysis

All statistical analyses were performed using GraphPad Prism 9.0 (GraphPad Software). Data are presented as mean ± standard deviation (s.d.) unless otherwise specified. For comparisons between two groups, two‐tailed unpaired Student's t‐test was used. For multiple group comparisons, one‐way ANOVA followed by Šidák's post‐hoc test was applied. P values less than 0.05 were considered statistically significant. Sample sizes (n), statistical methods, and P values are indicated in the corresponding figure legends. No data were excluded from analysis, and all experiments were repeated at least three times independently unless otherwise stated.

## Conflict of Interest

J.T. is an equity holder of Hangzhou LUCA Intelligent Technology Co. Ltd. Z.W.L. is currently an employee of Hangzhou LUCA Intelligent Technology Co. Ltd. The authors declare that they have no other known competing financial interests or personal relationships that could have appeared to influence the work reported in this paper.

## Author Contributions

R.M., J.L., W.W., and D.L. contributed equally to this work. J.Z., R.M. designed the experiments, R.M., J.Y.L., C.H., Q.Y.L., and Y.Z. performed the experiments. W.K.W., Z.W.L., and J.T. analyzed the data. R.M., J.Y.L., C.M., and J.Z. wrote the manuscript with help from all authors. D.L., H.P., Q.N., B.S., and J.Z. revised the manuscript. J.Z. and X.X.H. provided conceptual advice and supervised the work. All of the authors reviewed and approved the manuscript.

## Supporting information



Supporting Information

Supplemental Table 1

Supplemental Table 2

Supplemental Table 3

Supplemental Source data

## Data Availability

The deep sequencing data from this study have been submitted to the National Center for Biotechnology Information Sequence Read Archive database under accession number PRJNA1174101. Source data are provided with this paper.
