# Supplementary material for: Engineered Cas12j‐8 is a Versatile Platform for Multiplexed Genome Modulation in Mammalian Cells
Source: Adv Sci (Weinh). 2025 Jun 10;12(33):e02593. doi: 10.1002/advs.202502593 (PMC12412624; doi:10.1002/advs.202502593)
Supplement: Supplementary file 1 — Supporting Information [file ADVS-12-e02593-s005.docx]

**Engineered Cas12j-8 is a Versatile Platform for Multiplexed Genome Modulation in Mammalian Cells**

*Ru Meng, Jiayao Li, Wuke Wang, Dong Liang, Zhanwei Li, Cong Mao, Qingyang Li, Yu Zhang, Hao Chen, Jin Tang, Ping Hu, Qi Niu ^*^, Xingxu Huang ^*^, Bin Shen ^*^, Jun Zhang ^*^*

**Supplementary Figures**

**
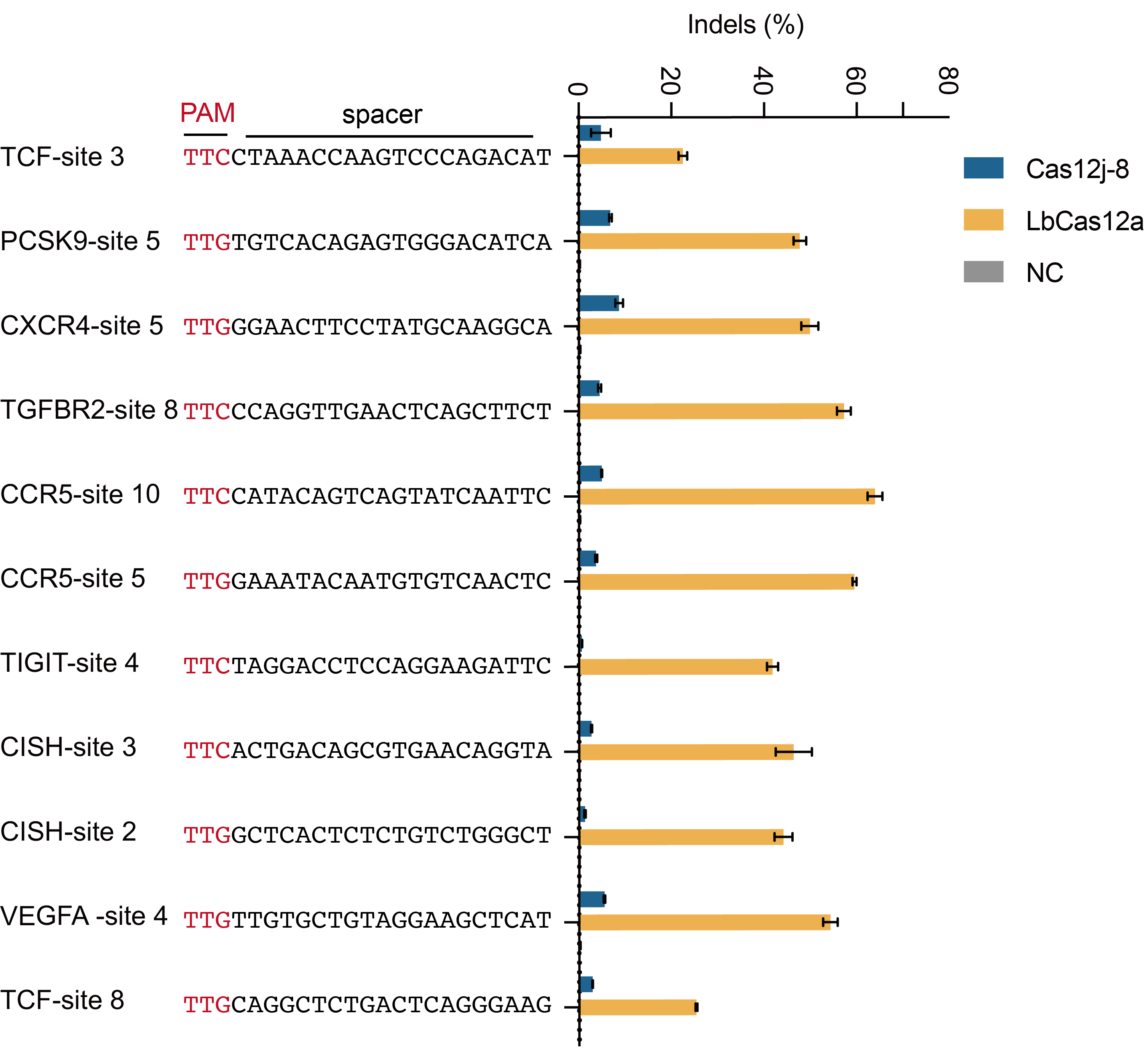
**

Figure S1. Gene editing efficiencies of Cas12j-8 and LbCas12a at endogenous genomic loci in HEK293T cells. The mean ± s.d. and individual data points are shown for n = 3 independent biological replicates.


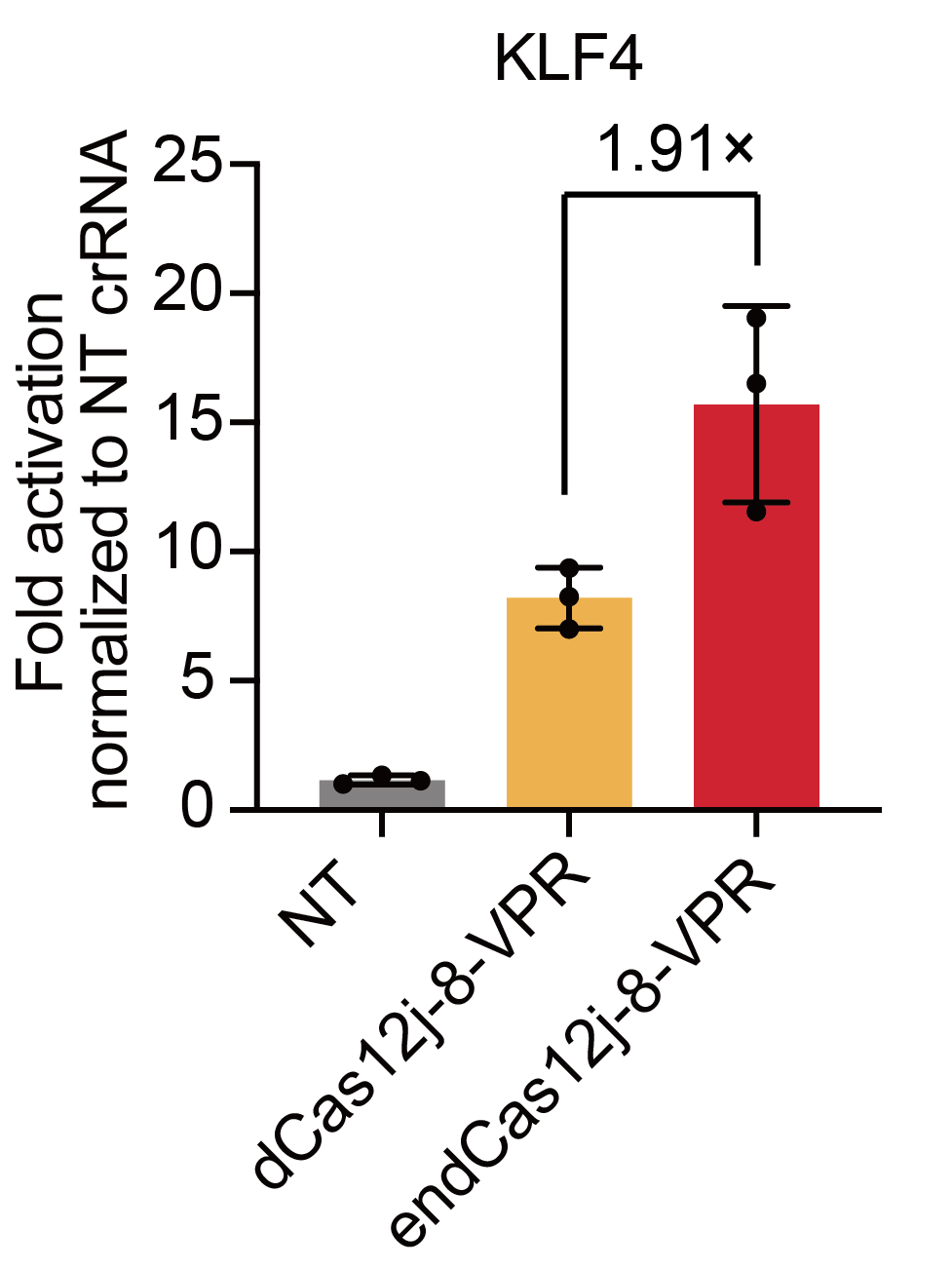


Figure S2. Gene activation of *KLF4* using dCas12j-8-VPR and endCas12j-8-VPR, measured using RT-qPCR in HEK293T cells. All data shown are fold activation of mRNA by normalizing to the mRNA expression using a non-targeting crRNA (NT). Data are shown as mean ± s.d., n = 3 independent experiments.

**
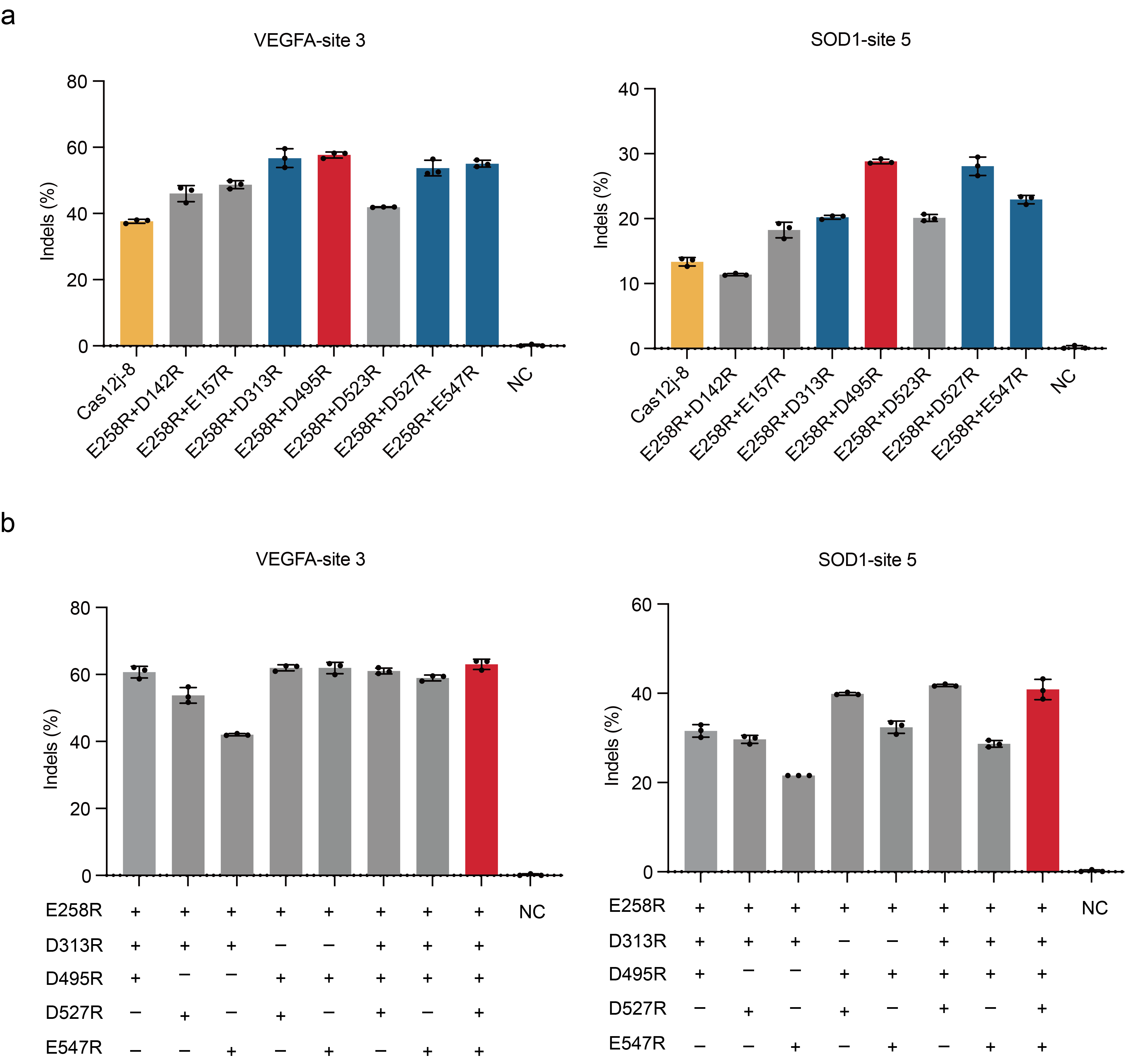
**

Figure S3. Comparison of gene editing efficiencies between Cas12j-8 and its mutants.

(a) Gene editing efficiencies of Cas12j-8 and its double mutants at endogenous genomic loci in HEK293T cells. Yellow indicates wild-type Cas12j-8, red denotes the optimal mutant, blue represents suboptimal mutations selected as candidates for the next round of mutations.

(b) Gene editing efficiencies of Cas12j-8 and its multiple mutants at endogenous genomic loci in HEK293T cells. Yellow indicates wild-type Cas12j-8, red denotes the optimal mutant. Data are shown as mean ± s.d., n = 3 independent experiments.

**
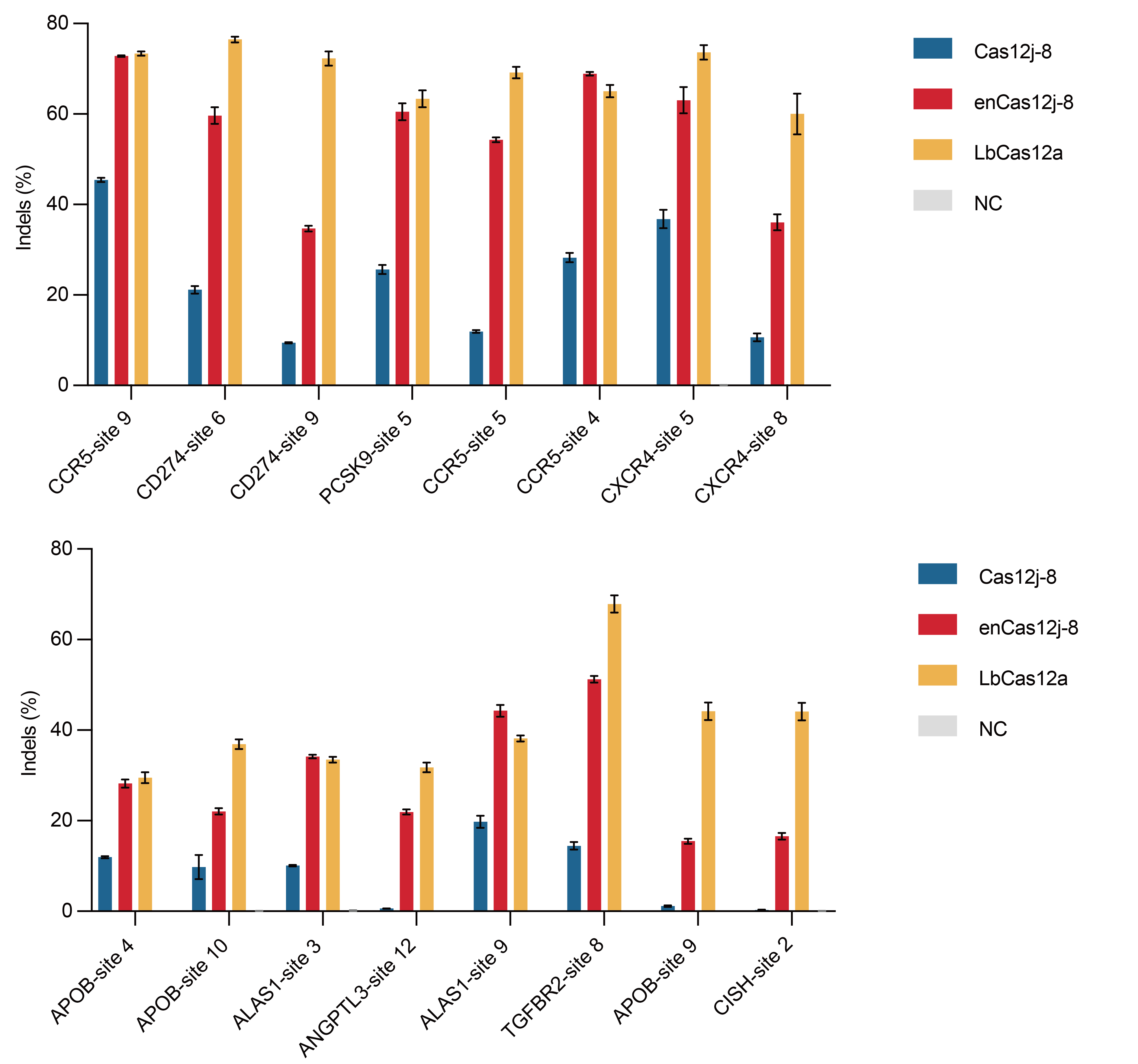
**

Figure S4. Gene editing efficiencies of Cas12j-8, enCas12j-8, and LbCas12a at endogenous genomic loci in HEK293T cells. The mean ± s.d. and individual data points are shown for n = 3 independent biological replicates.


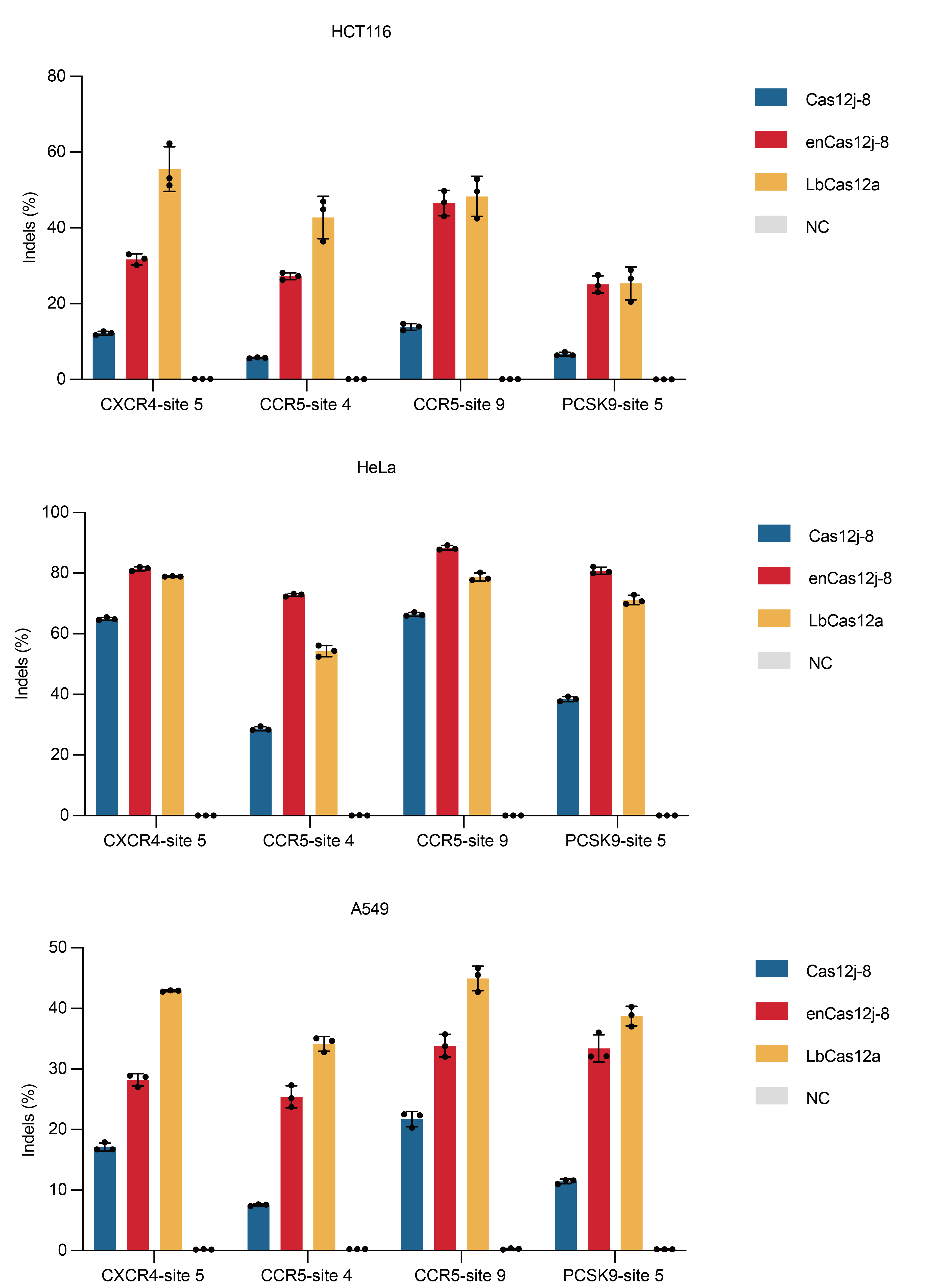


Figure S5. Comparison of genome editing efficiencies of Cas12j-8 and enCas12j-8 at endogenous loci in HCT116, HeLa and A549 cells. Data represent mean ± s.d. from n = 3 independent biological replicates.


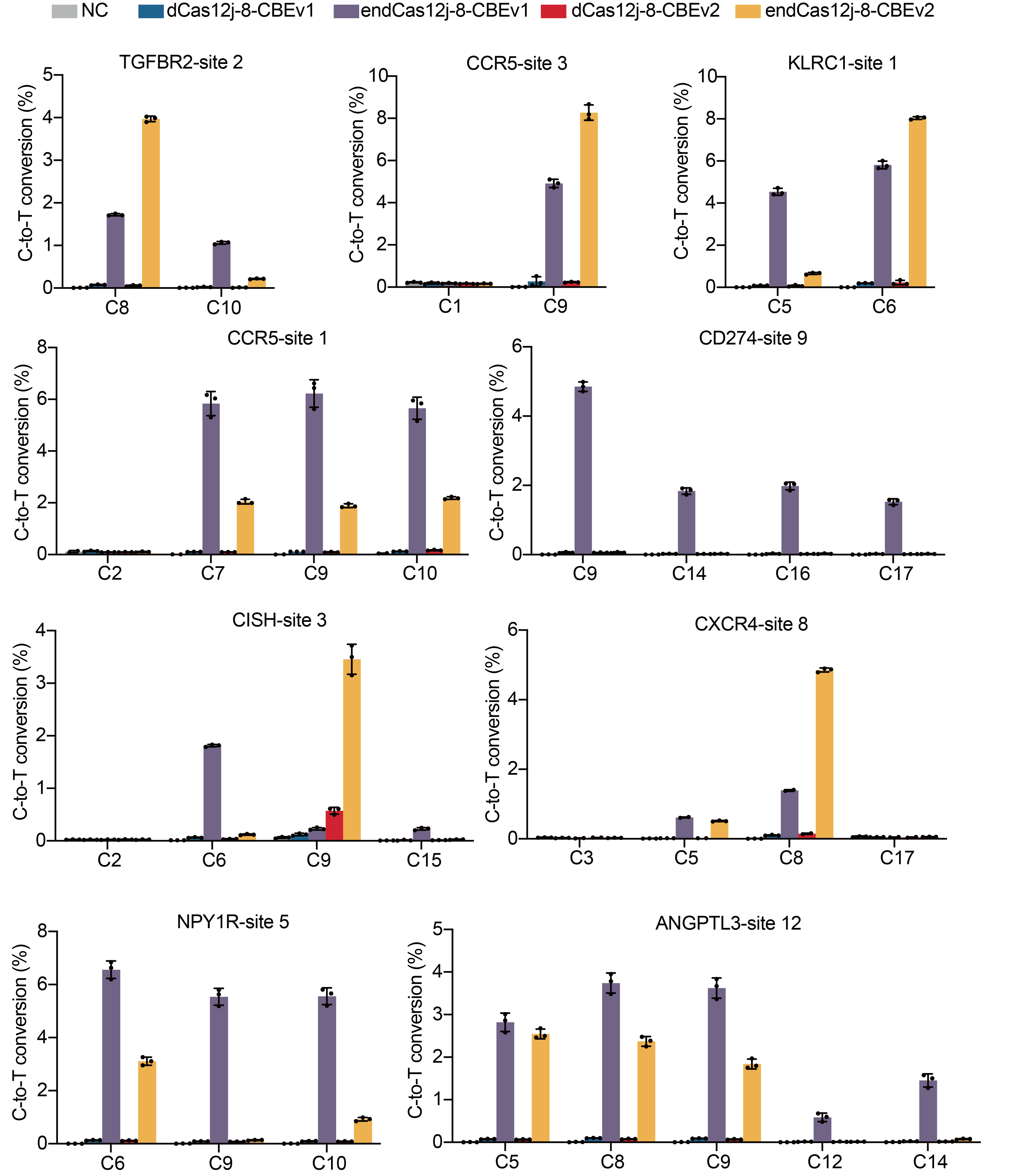


Figure S6. C-to-T conversion (%) of CBEv1 and CBEv2 at representative endogenous genomic loci. Data represent mean ± s.d., with individual data points from three independent experiments (n = 3) in HEK293T cells.


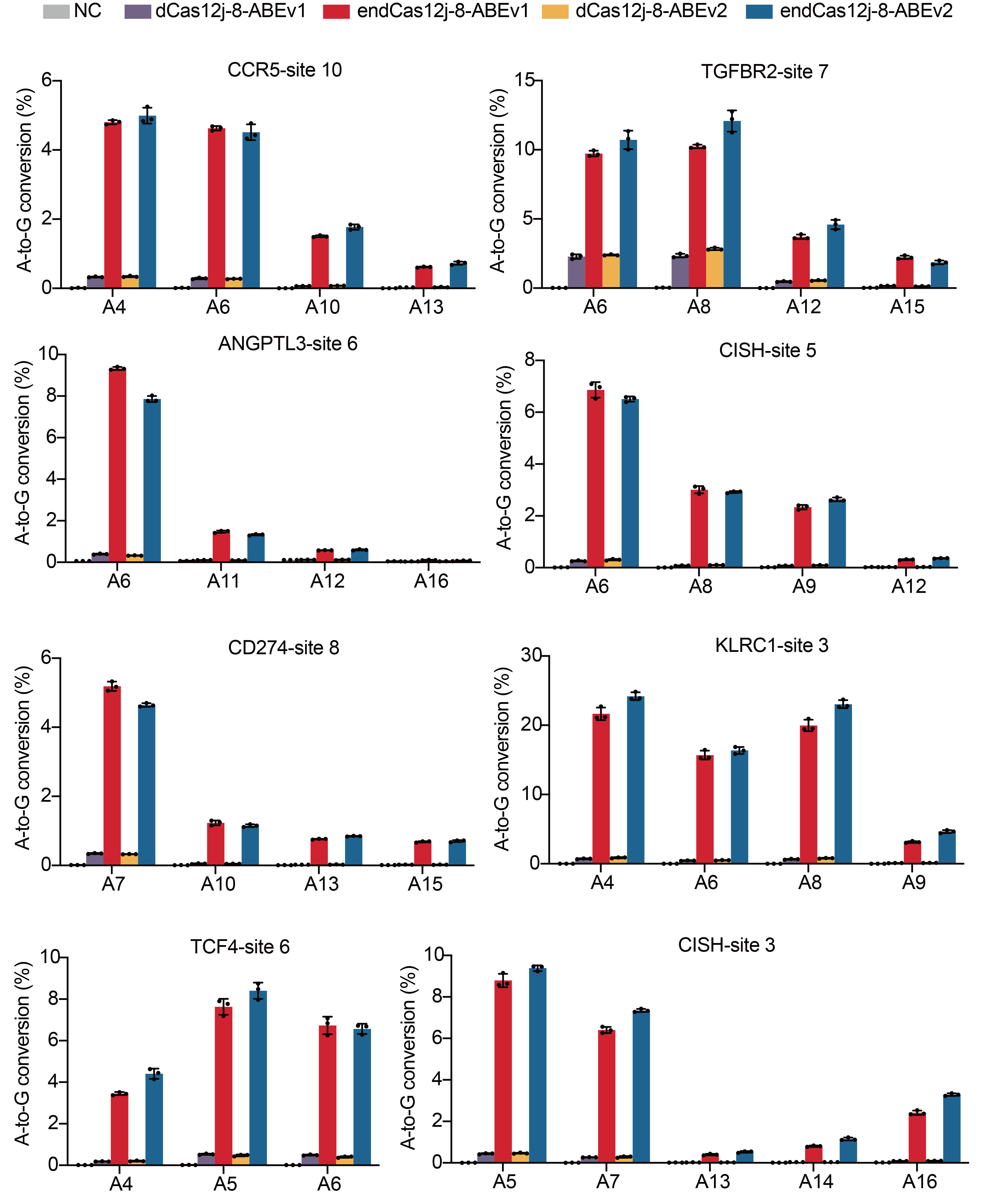


Figure S7. A-to-G conversion (%) of ABEv1 and ABEv2 at representative endogenous genomic loci. Data represent mean ± s.d., with individual data points from three independent experiments (n = 3) in HEK293T cells.


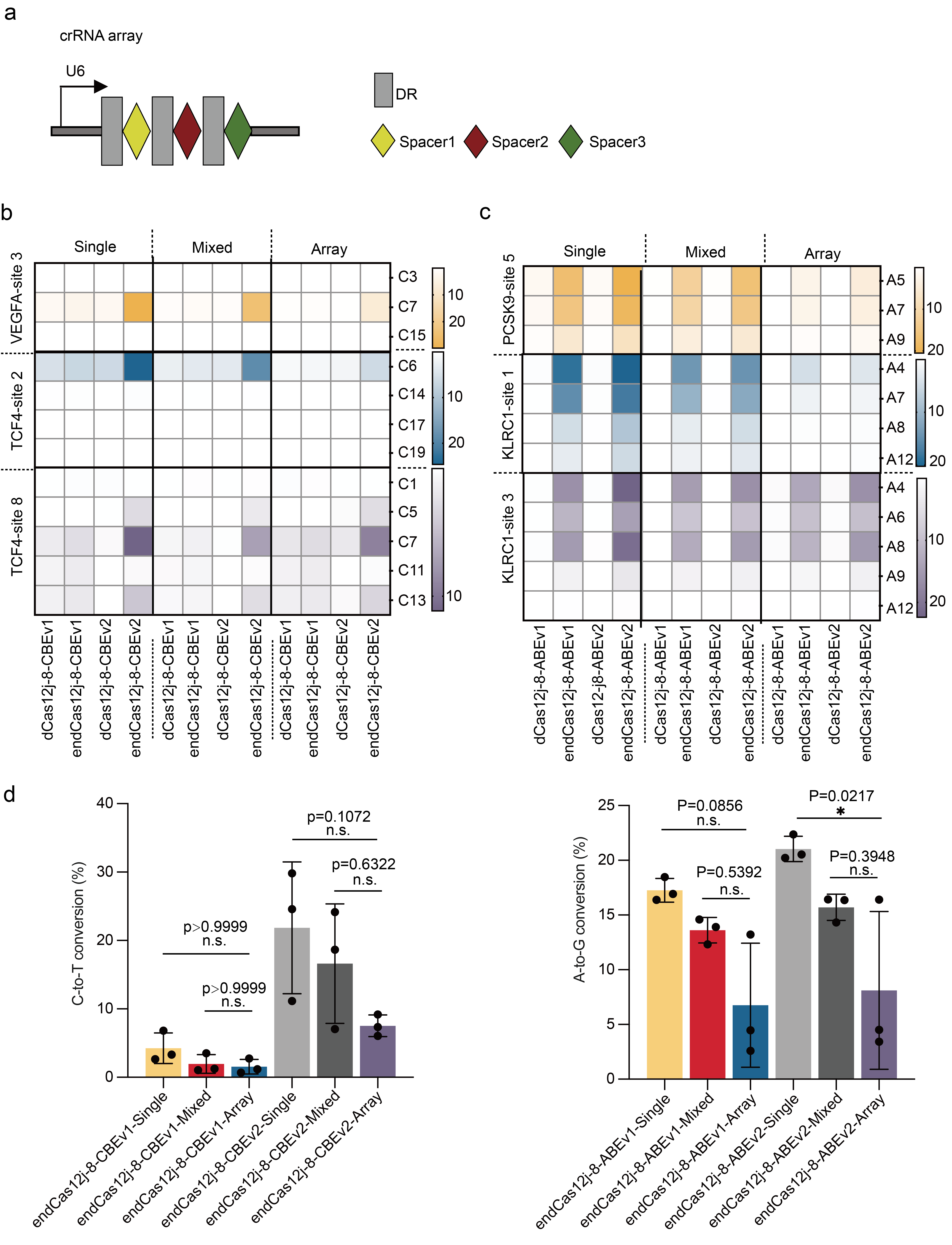


Figure S8. dCas12j-8-mediated multiplexed base editing in mammalian cells with non-HDV crRNA-array.

(a) Schematic of constructs used for multiplexed gene editing, featuring a non-HDV crRNA array with three crRNAs (different colored diamonds) separated by direct repeats (DR).

(b) C-to-T conversion (%) of CBEv1 and CBEv2 at three endogenous genomic loci simultaneously using a non-HDV crRNA array in HEK293T cells, compared to single crRNA editing and mixed-crRNA editing. Data represent the mean of three independent experiments.

(c) A-to-G conversion (%) of ABEv1 and ABEv2 at three endogenous genomic loci simultaneously using a non-HDV crRNA array in HEK293T cells, compared to single crRNA editing and mixed-crRNA editing. Data represent the mean of three independent experiments.

(d) Summary of editing efficiencies at the optimal editing position for each target site under single, pooled, or crRNA array conditions. Statistical significance was evaluated using one-way ANOVA followed by Šidák’s multiple comparisons test. n.s., not significant; *p < 0.05; **p < 0.01; ***p < 0.001; ****p < 0.0001.
